# Induced Native Phage Cocktails for Multi-microbial Activation Syndrome in Treatment-Resistant Illnesses

**DOI:** 10.7759/cureus.72587

**Published:** 2024-10-28

**Authors:** David A Jernigan

**Affiliations:** 1 Bioregulatory Medicine, Biologix Center for Optimum Health, Franklin, USA

**Keywords:** biofilm, biospectral emission sequencing, endotoxin, induced native phage therapy, inducen-ld/rf, inducen-res, lyme disease, multi-microbial activation syndrome, respiratory tract infections

## Abstract

The global population is plagued by chronic illnesses of many types due to, in part, the activation of virulence of many latent microbes leading to chronic multi-system illness, stimulating the new term, multi-microbial activation syndrome (MMAS). Treatment-resistant infections across the entire microbial spectrum are now largely untreatable using the previously successful pharmaceutical and natural medicine options. The heavy-handed methods of long-term chemotherapeutic antibiotics, antifungals, and antiviral drugs, whose adverse effects could be worse than the original illness, are presently essentially useless in the long run, as microbes have developed rapid adaptive mutations, becoming drug-resistant. The introduction of induced native phage therapy opened a completely new treatment category that has the potential to complement or replace aggressive drug therapies of many classes. Since its introduction, many advancements have been made to the technology. The most recent development in this new treatment genre is the ability to formulate induced native phage cocktails that use a wide spectrum of induction signatures within the formulation to stimulate various types of beneficial phages already living within the phageome of the body, to target a wide range of pathogenic microbes and associated advantageous physiological targets. The ability to address the MMAS commonly seen in various chronic illnesses simultaneously within the same formulation enables greater clinical outcomes without harm to the microbiome or to the tissues and systems of the body.

## Introduction and background

The global population is suffering from multi-microbial activation syndromes (MMAS) causing treatment-resistant, chronic illness and premature death, in contrast to past generations which were more often caused by an individual microbe [1].

The first report of antimicrobial resistance predated penicillin by four years [2]. It took less than 40 years for the global bacterial world to develop drug resistance to the once panacea, penicillin, and the speed of microbial mutation against each newly introduced chemical treatment is increasing [3]. Between 1990 and 2021, more than one million people died from drug-resistant infections each year, and this could increase to nearly two million by 2050. Around 92 million lives could be saved between 2025 and 2050 with wider access to better treatments for infections [4].

Hard lessons learned from history provide ample evidence that all lifeforms strive to adapt and survive, especially microbes. Pharmaceutical drug interventions are doomed to fail from the rapid mutation of microbes and due to the predictable adverse effects of damage to the patient, damage to the beneficial microbiome, and damage to the beneficial phageome, all of which contribute to the breakdown of the body's innate bioregulation of the pathogenicity of the microbes [5].

Though short-term improvements may seem to justify the use of chemical antibiotics and the new antimicrobial drugs in development that are expected to follow the same path, the true long-term consequences are predictably the development of chronic symptoms, more multi-drug resistant microbes, and degeneration as the microbiome balance is upset and the activation of multiple microbial populations lead to iatrogenic drug-induced symptoms, poor quality of life, and shortened lifespan of the patient [6,7].

Any treatment that does not work in harmony with the totality of the human organism is necessarily going to create predictable disharmony leading to systemic dysfunction and disease. It is this primary point of contention that leads one to appreciate the concept of inducing or stimulating beneficial phages (viruses) that are already a part of the human organism from birth to death, as in induced native phage therapy (INPT), to assist in the reduction or complete elimination of those populations of microbes that are over-populating and causing disease [8].

The now famous debate of the 1800s between the scientific giants and rivals Louis Pasteur and Antoine Béchamp, in which Pasteur argued that the microbe was the cause of disease while Béchamp argued that the terrain or the internal milieu of the body determined health or disease, has overwhelmingly been settled with the wisdom and knowledge gained since their day and age [9]. Both researchers were correct. Health is not the absence of microbes, yet when the healthy bioregulation, the internal milieu of the body is disrupted for whatever reason, microbes can cause disease. In our day and age, poor dietary choices, medical iatrogenic challenges, and environmental chemical and electromagnetic pollutants, not to mention the excessive use of agricultural chemicals corrupting the food chain and pharmaceutical drug-induced mutations of the microbiome of the planet, have created super-bugs with the disappearance of the classic forms of microbes [10,11].

The arsenals of natural and pharmaceutical medicines are no longer able to achieve the restoration of health enjoyed in the past. Microbial communities of the human body are opportunistically activating their virulence factors in unison in MMAS. It is a rare human in today’s world whose bioregulation is adequate to keep microbes in their body at bay, as would be expected in a person living a healthy, balanced life. Healthy bioregulation is harmed by introducing strong antimicrobials which function like an unselective bomb to the body’s microbiome. Although a patient may survive the treatment, overall bioregulation integrity and microbiome diversity are damaged beyond complete repair. More potent pharmaceuticals can only cause more harm, leading to a global quest to develop effective phage therapies [12,13].

Native phage is a general term for beneficial viruses that naturally exist in the human body from birth throughout life. The most widely recognized phages are bacteriophages, which use specific bacteria as a reproductive host, but there are also mycophages, which use fungi as a reproductive host, and other types of phages that use other microbes. All of the naturally occurring phages in the body are collectively known as the phageome, part of the well-recognized microbiome. Phages are the most dominant populations by far within the body’s microbiome. Researchers report identifying over 32,000 unique bacteriophage populations within healthy human intestines [14]. The native phageome helps the body’s immune system regulate the population growth of every type of microbe, whether it is a pathogenic infection or a beneficial bacterium, otherwise known as our friendly flora. Even beneficial bacteria would cause disease if they were to grow out of the regulatory control of the immune system and phages [15].

Contrasted to INPT, conventional phage therapy (CPT) is a compassionate use-only treatment at this time, existing solely within the pharmaceutical realm of medicine. CPT has been practiced openly in only a few locations around the world since its discovery by Felix d’Hérelle in 1917 [16]. Experimentally, biotech companies and universities are researching a third phage category of genetically modified or engineered phages [17], a topic which will not be covered herein, yet warrants mentioning.

CPT has sought to isolate and catalog many wild types of bacteriophages from bacteria-rich environments such as sewage, manure, soil, and other sources. These wild, non-native phages are kept in phage banks and historically one or more types of phages are matched to the specific person’s infection. When more than one type of phage is found that will kill the specific infection in a laboratory, it is called a phage cocktail, and multiple phages are given as treatment of a particular phage-resistant infection, where it is unlikely that a single phage type would be effective [18]. Historically, there are very few if any adverse effects of introducing these wild phages into the person with the infection [19]. The high specificity of wild phages used in CPT makes them unsuitable for patients with multiple infections or those with infections that cannot be identified by standard diagnostic testing [20]. There are concerns about the wild phages of CPT or the more advanced genetically modified phages such that the immune system will see these foreign phages as invaders or that they may cause unanticipated harm to the person. However, there is very little evidence of problems with the introduction of correctly tested wild phages [21].

We are conditioned to be fearful of viruses, although the vast majority of viruses in the body serve very important health benefits. As a matter of fact, recent research by Gregory et al. showed that a decrease in the number of viral (phage) populations in a person’s virome is associated with declining health and disease [22]. It appears that as we age, there is a decline in the number of these phage viruses in the virome of the body, matching the decline in a person’s health. The researchers tested the virome of almost 2000 people from 16 countries and identified 33,242 unique phage populations in the human gut, of which 97.7% were determined to be (native) bacteriophages [22].

INPT falls within the realm of natural or alternative medicine, which works with the natural design and function of the body in relation to the immune system and native phage populations bioregulation of every microbial population within the microbiome. INPT is produced using a proprietary technology, Biospectral Emission Sequencing (BES), which seeks to activate strategic lytic benefits from native or naturally occurring monovalent or polyvalent phages already living harmoniously in the human body, contrary to CPT which the reader will recall introduces wild phages into the body [23,24].

At its inception in 2019, just over a century after Felix d’Hérelle introduced bacteriophage therapy to tremendous resistance and controversy from the scientific community of his day [25], Jernigan et al. introduced the new technology of inducing native phages [26]. The working hypothesis was to determine if electromagnetic signatures could be sequenced in such a manner as to induce unspecific native phage populations, either monovalent or polyvalent, to eliminate a predetermined *Borrelia* strain specifically targeted with the induction process. Initial studies targeted only one type of *Borrelia* at a time, such as *Borrelia burgdorferi*, since it was unknown if more than one type of *Borrelia* or coinfection could be addressed at once without causing undue distress to the patient due to the anticipated Jarish-Herxheimer reaction [26].

As reported in the study by Jernigan et al., 20 (77%) of the 26 participants who initially tested positive for one or more strains of *Borrelia* infection tested negative after just a four-day treatment of INPT [26]. With modifications and prolonged treatment for four weeks, a total of 92% of participants tested negative, with many reporting mild to unprecedented clinical improvements. There were no reports of adverse reactions or Jarish-Herxheimer reactions in the group, due to the manner and the speed at which phages kill the *Borrelia*, which as a bacterium have no endotoxins to cause a reaction [27]. To document long-term elimination of the infection, in the 20 patients who tested negative, eight were tested by Jernigan et al. a third time, between eight and 24 weeks after their negative test without receiving further Inducen-LD or antibiotic treatment [26]. Of these eight patients, seven (88%) remained negative for the indications of their initial strain of *Borrelia*, demonstrating long-term clearance of the *Borrelia* without rebound growth of infection below the sensitivity of the testing to detect. The study had many limitations due to the fact that it was started the week before the COVID-19 pandemic was declared, and ended in 2021 [26].

According to data collected during the study by Jernigan et al. [26], the first author of which is also the author of the current review, a few patients tested as having multiple strains of *Borrelia* (Unpublished data; Available on request to the author). When only one strain was targeted with INPT, only that strain was negative upon retesting with the other strain persisting. When the other strain was subsequently targeted, it too was tested as negative post treatment, as was the initial strain. This finding demonstrates the specificity of the phage-induction process and the need to specifically target the correct strain when using INPT.

## Review

Induced native phage cocktails

In response to the universal nature of multi-microbial populations all being actively virulent at the same time in most systemic, chronic illnesses, it became apparent that until the terrain of the body could be restored, the multitude of primary virulent infections needed to be successfully addressed. The terrain was universally damaged by multiple challenges, each of which needed to be addressed. The issues with the structural and functional integrity of the body set the stage for multiple infections to arise, thereby complicating the situation [28].

Ongoing clinical studies have shown overwhelming positive clinical results while using many different INPT electromagnetic signatures at the same time (Principle investigator: David A. Jernigan, Title of Study: A Quality of Life Outcomes Study of Patients with Chronic, Non-Responsive Genetic or Acquired Illnesses and Pain Syndromes Using the Diagnostic and Treatment Technologies of Chiropractic Medicine and American Biological/Bioregulatory Medicine (CMABM). Date of initiation: March 9, 2020; Title of Study: An Observational Study of the Dietary Supplement, Induced Native Phage Therapy, Combined with the Treatment Philosophies and Techniques of Chiropractic Medicine and American Biological/Bioregulatory Medicine (CMABM), Date of initiation: February 22, 2024). After over 100,000 doses administered, no adverse reactions or uncontrollable Jarisch-Herxheimer reactions have occurred, demonstrating INPT is safe and effective. Dosages were self-administered in individually dosed 2 ml sterile solution in glass ampules. The daily dosage recommendation was one ampule morning and evening orally, swish and swallow in a naturally clean mouth, typically for one to six weeks.

With the development of induced native phage cocktails, each of the electromagnetic signatures for targeting a native phage population to reduce or eliminate a specific infection can exist and persist within the same formulation and act independently of the other induction signatures. Conventional antibiotics work in a similar manner, reducing or eliminating a broad spectrum of bacteria, unfortunately including beneficial bacteria. In native phage cocktails, only the targeted infections are reduced or eliminated, while leaving beneficial bacteria unharmed. Due to the precision targeting of only the pathogenic microbes, no disruption of the microbiome was observed, and no secondary yeast overgrowth has been reported. All of this is accomplished with no detectable involvement of the immune system or bioregulation systems of the body unless one comprehends that native phages are indeed an integral, though unappreciated, component of the human body in health and disease. INPT works with the natural bioregulation of the body in a non-pharmaceutical manner. The clinical physician is often faced with patients presenting with symptoms of unspecified infections such as a respiratory infection. Conventional drug therapies, such as antibiotics, are given with the understanding that they are broad-spectrum, so the exact types of infection will likely be addressed. Induced native phage cocktails were formulated with that in mind, being very specific to the types of typical and atypical infections targeted, yet without the harmful effects to the patient's microbiome and without the predictable antibiotic-induced damage of mitochondria.

Examples of MMAS illness targeted by induced native phage cocktail formulations are those designed to address respiratory infections called Inducen-Res and Inducen-LD/RF to address the many infections and coinfections of Lyme disease and relapsing fever (*Borrelia *infections).

The microbes that have been specifically targeted with Inducen-Res (PhagenCorp, LLC, Sarasota, Florida, United States), are *Bordetella holmesii, Bordetella pertussis, Burkholderia cepacian* + lipopolysaccharides (LPS)/endotoxin, *Burkholderia pseudomallei +endotoxin/LPS, Haemophilus influenzae, Moraxella catarrhalis, Mycoplasma pneumoniae, Mycoplasma hominis, Chlamydophila pneumoniae (Chlamydia pneumoniae), and Legionella pneumophila, Mycobacteria Avium complex, Mycobacterium abcessus, Mycobacterium kansasii, Mycobacterium intracellularis, Mycobacterium tuberculosis*, multi drug-resistant *M. tuberculosis*, LPS + endo/exotoxins, *Prevotella melaninogenica, Pseudomonas aeruginosa, Pseudomonas fluorescens*, methicillin-resistant *Staphylococcus aureus* (MRSA), *S. aureus, Streptococcus pneumoniae, Streptococcus pyogenes, Enterococcus faecalis, Streptobacillus moniliformis, Streptococcus mutans, Streptococcus agalactiae, Stenotrophmonas maltophilia*, *Candida *spp., *Aspergillus fumigatus, Coccidioides* spp., *Aspergilla* *niger*, *Pneumocystis carnii*, and *Protomyxzoa rheumatica.*

The microbes that have been specifically targeted with Inducen-LD/RF (PhagenCorp, LLC) are *Babesia bigemina, Babesia bovis, Babesia canis, Babesia cati, Babesia divergens, Babesia duncani, Babesia felis, Babesia gibsoni, Babesia herpailuri, Babesia jakimoni, Babesia major, Babesia microti, Babesia ovate, Babesia pantherae, Bartonella alsaticca, Bartonella arupensis, Bartonella baciliformis, Bartonella berkhoffii, Bartonella birtlesii, Bartonella bovis, Bartonella capreoli, Bartonella clarridgeiae, Bartonella doshiae, Bartonella elizabethae, Bartonella grahamii, Bartonella henselae, Bartonella koehlerae, Bartonella melophagi, Bartonella muris, Bartonella peromyscus, Bartonella quintana, Bartonella rochalimae, Bartonella schoenbuchii, Bartonella talpae, Bartonella taylorii, Bartonella tribocorum, Bartonella washoensis, Bartonella vinsonii, Borrelia afzelii, Borrelia berbera, Borrelia bisseti, Borrelia burgdorferi, Borrelia carteri, Borrelia caucasica, Borrelia crocidurae, Borrelia duttonii, Borrelia garinii, Borrelia hermsii, Borrelia hispanica, Borrelia kochis, Borrelia miyamotoi, Borrelia morganii, Borrelia novyi, Borrelia parkeri, Borrelia persica, Borrelia recurrentis, Borrelia tillae, Borrelia turicatae, Borrelia valaisiana, Borrelia venezuelensis, Borrelia vincentii*, *Leptospira*, human monocytic and granulocytic *Ehrlichia, Ricketsia rickesii, Moraxella osloensis, Protomyxzoa rheumatica, Mycoplasma fermentans* incognitus, *Mycoplasma pneumoniae, Mycoplasma hominus, and Mycoplasma genitalium*.

Each strain and species must be specifically targeted by INPT; therefore, any infection that is not included in these formulations will not be addressed and must be customized directly. It is impossible to induce a native phage to target an infection or strain which does not exist within the person being treated. The induction signatures within the native phage cocktails which are not relevant dissipate harmlessly. Because of this fact, native phage cocktails have been developed that target over 100 types of infections in a single formulation, each of which typically or atypically are seen in a given disease process. In other words, although a patient may not have 100 types of problematic infections, only the types of infections they do have will be targeted from among those included in the INPT cocktail. The extraneous induction signatures appear to dissipate harmlessly. Prior to the development of induced native phage cocktails, the targeting of the primary infection, when effective, left a vacancy for opportunistic infections to rise to the level of priority, especially when the internal milieu had not been improved. Not only can the specific numerous primary infections and coinfections be targeted with INPT cocktails but the microbial toxins, such as endotoxins, exotoxins, and LPS can also be targeted at the same time. Many of the targeted microbes are known to produce formidable biofilms; however, fluorescing microscopy studies have verified the effectiveness of phages in dissolving biofilms [29]. Research has shown that even when CPT (wild phages) was only partially successful, it still decreased biofilms and increased the effectiveness of more classic antibacterial treatments [30].

When INPT is successful, the rapid killing of targeted microbes raises concerns of increased release of microbial endotoxins and LPS, both of which are highly proinflammatory chemicals. Due to the way native phages kill their targeted microbes, this is rarely seen in clinical practice. Using the techniques of INPT, many induced native phage cocktails have been formulated to also induce native phages to directly target the endotoxins and LPS substances from the microbes and their biofilm. The ability to target the microbes and their toxins enhances the gentleness of this form of treatment and has been seen to improve subjective symptom relief [31]. The goal of inducing native phages to eliminate the infections rapidly and gently, and also dissolve the toxins appears to be effective upon various unpublished post-treatment laboratory testing and favorable clinical results [32].

It is important that native and conventional phages can break through biofilms. Microbial biofilms often contain the primary substances of pathogenicity beyond the actual microbial infection, so any pharmaceutical treatment that targets only the infection may result in moderate symptomatic relief, yet not change the underlying disease process due to the residual biofilms [33].

Over 100,000 doses of INPT formulations have been given under research protocols for a wide range of conditions with no reported adverse effects and none to minimal Jarisch-Herxheimer reactions when combined with comprehensive therapies [34]. A predictable observation is that when the infection is caught early, the induced native phage cocktails often relieve the condition within 12-48 hours of bi-daily treatments although it is advisable to continue the treatment for a minimum of five days. When the infection is complicated and chronic, positive clinical response can often be seen with one to six weeks of bi-daily treatment. If no response is seen, it is often the case that the wrong microbes are being targeted and deeper testing must be done to identify the primary infection.

Limitations of INPT and recommendations

INPT is a new category in the healthcare arsenal of treatments, whose proposed mechanism of action is largely based upon an established technology, BES, with 30 years of clinical use. The working hypothesis that BES could be sequenced for the express purpose of causing unknown but willing native phage populations to specifically turn virulent, possibly expressing endolysins against the targeted infection is substantiated as safe and effective. Further support is gained by the reverse engineering of cause and effect, yet limitations of documenting phage induction are obvious since the harnessing of virtually invisible phages of unknown types from the collective phageome is impossible to verify in vivo [33].

To minimize the worsening of patient symptoms caused by endotoxins and exotoxins from various strains of infection, BES testing was sequenced to specifically target these toxins, such as LPS. These electromagnetic signatures were added to the INPT signatures to be a part of the final treatment solution. Other alternative supplementation is often used to facilitate the efficient removal of toxins that are not eliminated by phage action. The concern that inducing specific native phages to eliminate the targeted infections might inadvertently activate bacterial pathogenicity via increased production of toxins, has not been observed in the four years of treatment of 1,736 study participants [26,34].

It has been documented that phage can penetrate cells and microbial biofilms to reach their target microbes**. **Phages produce enzymes, primarily depolymerase and lysins in their capsid, that have been shown to dissolve biofilms [32,35].

Although the in vitro effectiveness of wild phages can be observed in CPT, the effectiveness of INPT can only be seen by in vivo clinical results, due to the fact that INPT enlists the aid of native phages and therefore cannot be visualized in a petri dish setting. The actual visualization of the phages in action within the patient, whether CPT or INPT, is unobservable beyond the positive outcome. However, BES testing has over 30 years of development and clinical use, lending credence to the compelling clinical and microbial lab testing results of sequencing of electromagnetic signatures for the express purpose of inducing native phages [23].

INPT cocktails will only target the specific microbes included in the formulation. It is common to see a poor response to treatment when the primary infection was not properly identified and was not included in the formulation of the Induced Native Phage cocktail. In CPT (wild), a limitation is that phage resistance emerges quickly, necessitating the use of phage cocktails of several types of wild phages laboratory tested to be able to eliminate the patient's infection to slow resistance formation [20]. The reliance on the inherent impulse of the wild phages used in CPT to seek out and eliminate the targeted infection is different than inducing native phages whose impulse to eliminate the targeted infection can be modified dynamically to adapt around bacterial resistance. A similar limitation is also seen when the native phages are repelled by the targeted infection. Targeted microbes mount defense mechanisms against native or wild phages, such as the bacterial enzyme CRISPR-cas9 and retrons [36,37].

Various bacterial populations are more successful in their defenses against phage infections. Conversely, not every patient has the same range of native phage populations that can be induced. The more antibiotics a person has taken, and the more insults to their native phage populations from other pharmaceutical and antiviral treatments, the fewer phage populations there are to induce, reducing the effectiveness of treatment [22]. However, it is rare that the induction of native phages is completely ineffective at eliminating some of the targeted microbes; beneficial effects are usually seen that are as good or better than conventional chemotherapeutic treatment options.

Limitations can occasionally be seen with the production of microbial biofilms, which are structured communities of one or more types of bacteria encased in a self-produced polymeric matrix. These biofilms can be soft to hard structures, such as bacterial plaque on teeth. Phages can disrupt and penetrate biofilms in order to reach the bacteria, making them potent agents against biofilm-associated, multi-drug-resistant infections [38].

For this reason, it is good to be able to utilize other modalities and treatments to assist the native phages such as strategic oral biofilm degrading enzyme supplements, intravenous methylene blue, and photodynamic therapy [39,40] The native phage cocktails must be repeated two to three times per day for weeks to pressure the native phages to stay in the lytic state, a state that is not natural to phages, which normally exist in their lysogenic phase, the rate-limited parasitic activities of their bacterial reproductive host. These INPT formulations have been modified to include many induction signatures for each targeted microbe in the formulation to increase the energy of induction and to help ensure they work for each patient who may not have the same native phage populations. In INPT, the enlisting of known monovalent or unknown polyvalent native phage populations of potentially many types with the goal of eliminating the targeted bacteria through direct lytic attack, could theoretically also cause bacterial cells to commit suicide, also known as “altruistic cell suicide”, thereby reducing the spread of phages and protecting the overall bacterial population [41].

Independent blinded studies are needed for various types of infections and stages of infections in order to increase acceptance of INPT and induced native phage cocktails by the healthcare community.

## Conclusions

MMAS has replaced the idea of a single infection in most if not all chronic illnesses. Induced native phage cocktails, contrasted to wild phage or genetically modified phage therapy, offer a more natural solution to precision targeting of the key infections commonly seen in such conditions as Lyme disease, chronic urinary tract infections, chronic respiratory tract infections, chronic arthritis, chronic inflammatory response syndrome, and the many other chronic infections, as well as systemic autoimmune illnesses involving infections and their toxins.

Native phages are referred to as the phageome and are an integral part of the microbiome of the healthy human body. The strategic activation of naturally occurring phages to our benefit is potentially superior to introducing wild phages into the body. The future of CPT appears to be set for genetically engineered phages to enter the healing arsenal. While this could possibly have significant advantages and lead to incredible results, these technologies are not currently available and will require many years of testing before they will be approved. INPT cocktails can be customized to the individual and as new infections take center stage, new INPT cocktail formulations can be easily developed to work in harmony with the body’s natural design and function.
